# Protein Expression Profile of Twenty-Week-Old Diabetic db/db and Non-Diabetic Mice Livers: A Proteomic and Bioinformatic Analysis

**DOI:** 10.3390/biom8020035

**Published:** 2018-06-01

**Authors:** Juan Manuel Guzmán-Flores, Elsa Cristina Flores-Pérez, Magdalena Hernández-Ortiz, Katya Vargas-Ortiz, Joel Ramírez-Emiliano, Sergio Encarnación-Guevara, Victoriano Pérez-Vázquez

**Affiliations:** 1 Departamento de Ciencias Médicas, División de Ciencias de la Salud, Campus León, Universidad de Guanajuato, León, Guanajuato 37320, Mexico; juan.guzman@cualtos.udg.mx (J.M.G.-F.); elsi28@hotmal.com (E.C.F.-P.); kavati75@hotmail.com (K.V.-O.); joelre@ugto.com (J.R.-E.); 2 Departamento de Clínicas, División de Ciencias Biomédicas, Centro Universitario de los Altos, Universidad de Guadalajara, Tepatitlán, Jalisco 47600, Mexico; 3 Centro de Ciencias Genómicas, Universidad Nacional Autónoma de México, Cuernavaca, Morelos 62210, Mexico; magdini@gmail.com (M.H.-O.); encarnac@ccg.unam.mx (S.E.-G.)

**Keywords:** bioinformatics, diabetes, mouse, obesity, proteomics

## Abstract

Type 2 diabetes mellitus is characterized by insulin resistance in the liver. Insulin is not only involved in carbohydrate metabolism, it also regulates protein synthesis. This work describes the expression of proteins in the liver of a diabetic mouse and identifies the metabolic pathways involved. Twenty-week-old diabetic db/db mice were hepatectomized, after which proteins were separated by 2D-Polyacrylamide Gel Electrophoresis (2D-PAGE). Spots varying in intensity were analyzed using mass spectrometry, and biological function was assigned by the Database for Annotation, Visualization and Integrated Discovery (DAVID) software. A differential expression of 26 proteins was identified; among these were arginase-1, pyruvate carboxylase, peroxiredoxin-1, regucalcin, and sorbitol dehydrogenase. Bioinformatics analysis indicated that many of these proteins are mitochondrial and participate in metabolic pathways, such as the citrate cycle, the fructose and mannose metabolism, and glycolysis or gluconeogenesis. In addition, these proteins are related to oxidation–reduction reactions and molecular function of vitamin binding and amino acid metabolism. In conclusion, the proteomic profile of the liver of diabetic mouse db/db exhibited mainly alterations in the metabolism of carbohydrates and nitrogen. These differences illustrate the heterogeneity of diabetes in its different stages and under different conditions and highlights the need to improve treatments for this disease.

## 1. Introduction

Type 2 Diabetes Mellitus (T2DM) is one of the fastest growing diseases, currently reaching pandemic levels. Its development is closely associated with obesity [1,2]. Physiological alterations resulting from high calorie intake do not only affect just a few key proteins or other functionally important biomolecules, but homeostasis is also guaranteed by the robustness of molecular networks [3,4]. It is thus conceivable that identification of deregulated pathways and networks is a fruitful approach as a starting point for the analyses of molecular mechanisms and validation of potential disease biomarkers.

The search for novel proteins or pathways linked to complex human diseases has been facilitated by proteomic technologies. Several proteomic studies have provided global proteome profiles of tissue and organs from T2DM patients or mouse (*Mus musculus*) models, identifying protein profiles associated with the pathogenesis of T2DM and its complications in tissues, such as in the pancreas [5], the adipose tissue [6], in muscles [7] and in the liver [8,9,10,11,12,13,14]. The liver is involved in the natural history of the ongoing epidemics of T2DM due to its participation in increased glucose production and dysregulated lipoprotein metabolism [15]. Additionally, several proteins that are exclusively or predominantly secreted from the liver are now known to directly affect glucose and lipid metabolism. Impaired insulin signaling in the liver and adipose tissue results in increased endogenous glucose production, which leads to hyperglycemia [15]. However, the mechanisms involved in the pathophysiology of this disease have not yet been clarified, and there could be other proteins involved in this process.

Due to the ethical and technical drawbacks of conducting studies in humans, testing in animal models for biomedical research is a good option. For the realization of this work, we use the db/db mouse, which is characterized by a mutation in the leptin receptor. This alteration results in hyperphagia, obesity (at about the age of 4 weeks), hyperinsulinemia (at about the age of 2 weeks), and insulin resistance for a later development of hyperglycemia (4–8 weeks); usually these mice do not live beyond 32–40 weeks [16].

Furthermore, age is associated with immunosenescence and by a chronic inflammatory state which contributes to multiple diseases [17]. Specifically, over time, all tissues, in particular the adipose and liver, exhibit important alterations in global gene expression profiles, accompanied by changes in the inflammatory state, cellular stress, fibrosis, altered capacity for apoptosis, xenobiotic metabolism, normal cell-cycle control, and DNA replication. Therefore, these changes influence the development of many diseases, including diabetes [18,19]. In a previous study, our research team reported alterations in proteins and metabolic pathways related to carbohydrates and lipids in 10-week-old mice [14]. Given these evidences, we anticipated that high blood glucose in db/db mice would be sufficient to predict a change in expression profile of hepatic proteins, which are related to diabetes complications. Therefore, the aim of this study was to analyze the changes in protein expression of the 20-week-old diabetic mouse liver.

## 2. Results

To confirm the conditions of diabetic and control mice, blood glucose was determined. At the time of sacrifice, blood glucose levels in db/db mice were higher than in the wild-type mice (488.2 ± 147 vs. 152 ± 22.5 mg/mL, *p* < 0.01).

**Figure 1:**
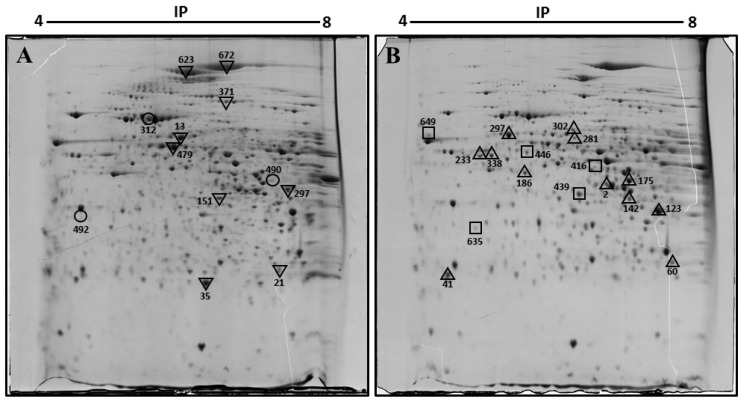
Representative two-dimensional electrophoresis image of the liver of control (**A**) and diabetic obese db/db (**B**) mouse. Proteins were separated by isoelectric focusing (IEF) in the first dimension, pH 4–8 Linear, then by size in the second dimension. Spots found with differential expression are marked in the gels. In (**A**): **▽**, diminished proteins in db/db mice. **○**, disappeared proteins in db/db mice. In (**B**): **△**, increased proteins in db/db mice. **□**, appeared proteins in db/db mice liver. Spots 233 and 338 were the same protein, keratin, type I cytoskeletal 18.

To identify the changes in protein expression associated with diabetes and obesity, we compared the expression profile of proteins in the liver of diabetic obese db/db mice versus non-diabetic mice using two-dimensional gel electrophoresis (2-DE). Three samples from liver homogenates obtained from each group were included for proteome analysis. Figure 1 displays representative 2D gel maps of liver proteins obtained from two groups. A total of approximately 600 spots were resolved on 2D gels. Spot volumes (area x intensity) were determined as a measure of protein expression using the ImageMaster 2D Platinum software. Spots that showed a 1.5-fold increase or decrease were included in the analysis, and proteins that are present in appreciable amounts were picked for identification by mass spectrometry (Figure 1).

The spots were excised from the gel, digested with trypsin, and identified by mass spectrometry. The results are shown in Table 1. Comprehensive analysis revealed that 29 spots registered a significant difference in diabetic hepatic tissue, 26 proteins were identified. Note that two spots were identified as the same protein, and that the identity of 3 spots could not be obtained. Of the 26 proteins identified, 12 protein spots were up-regulated and 8 were down-regulated in the liver samples of db/db mice compared with those of healthy mice. In addition, three spots appeared de novo, and in three other spots, expression was no longer detected in db/db mice.

**Figure 2:**
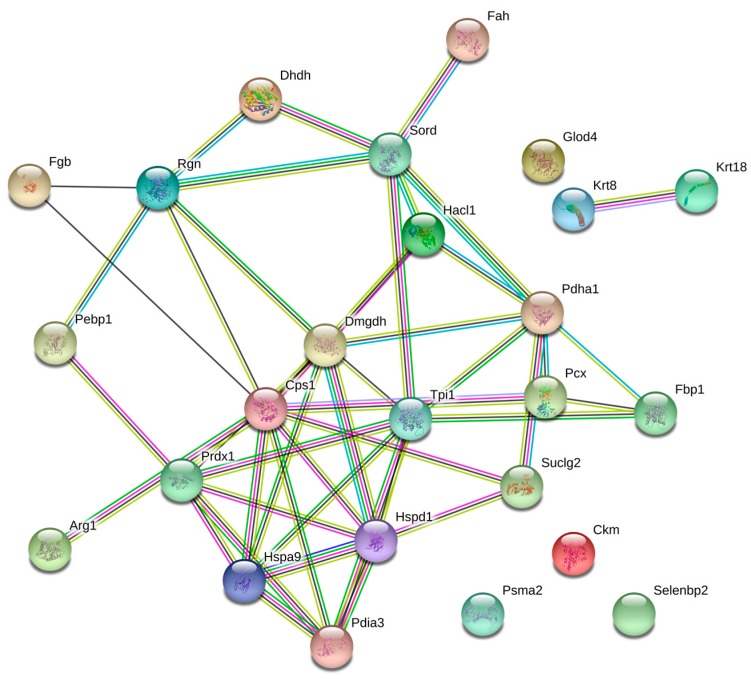
Network of differentially expressed proteins. The nodes stand for differentially expressed genes, and the lines stand for the interactions between two proteins.

**Figure 3:**
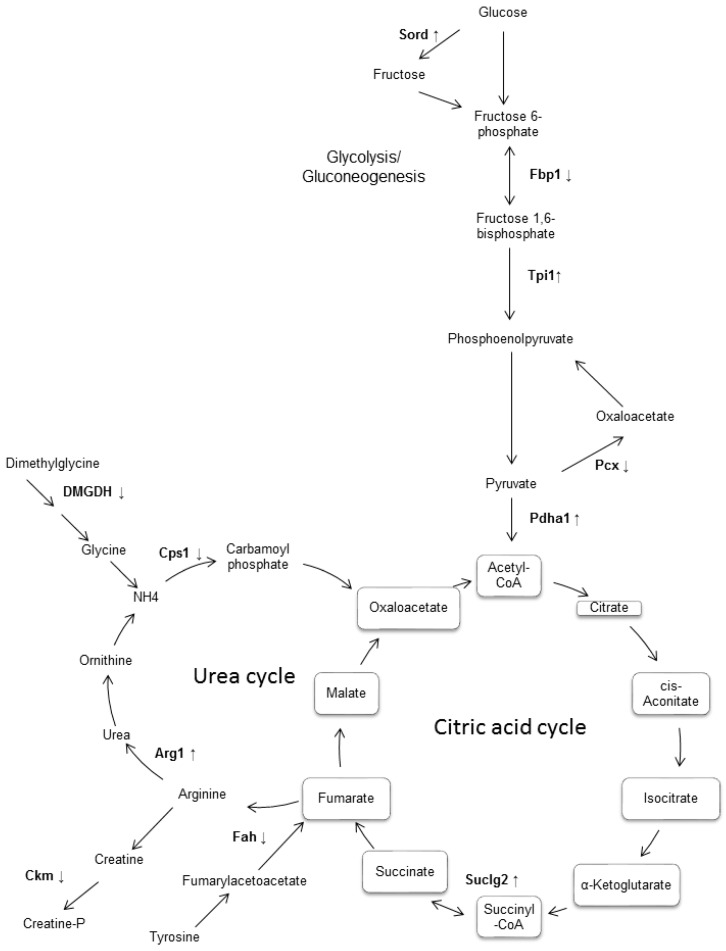
Representative diagram of the main metabolic pathways which involved some of differentially expressed proteins in the liver of db/db diabetic mouse. It was observed that most of the differentially expressed enzymes were found to participate in the metabolic pathways of gluconeogenesis/glycolysis, as well as in the metabolism of fructose and in the citric acid cycle. Although no enrichment of the urea cycle was observed, this was included because it binds both to the citric acid cycle and to the metabolic pathways of the enriched amino acids found. The enzymes that were found differentially expressed in this work are shown in bold, with an arrow indicating if it was increased or decreased.

To achieve a better understanding of the involvement of these proteins in the biological processes related to the pathophysiology of diabetes, these molecules were uploaded in the Search Tool for the Retrieval of Interacting Genes/Proteins (STRING) database, with the purpose of inferring a network from these proteins, which is shown in Figure 2. Likewise, the altered proteins were classified using Gene Ontology (GO) database and Kyoto Encyclopedia of Genes and Genomes Pathway (KEEG) pathway. For this purpose, the Database for Annotation, Visualization and Integrated Discovery (DAVID) software was used. Classification of proteins by biological process indicated that most of proteins were associated with glucose metabolism, cellular amide metabolism, glutamine family amino acid metabolism, carboxylic acid catabolism, and oxidation-reduction processes (Figure 3).

Protein classification by molecular function revealed that most proteins were implicated in purine nucleoside binding and vitamin binding. Classification of these proteins using GO database showed that differentially expressed proteins comprised mitochondrial proteins, especially matrix. Finally, to investigate pathways which were differentially expressed in diabetic mice livers, the KEEG pathway was used to sort and showed that most of these molecules are involved in the tricarboxylic acid cycle, the fructose and mannose metabolism, the arginine and proline metabolism, and glycolysis or gluconeogenesis (Table 2).

## 3. Discussion

This paper describes alterations found in the expression of 26 proteins identified in the liver of the diabetic mouse (db/db), which are associated with the metabolism of carbohydrates and amino acids, in addition to oxidation–reduction reactions. The findings in diabetic mice suggest that glucose metabolism is impaired in the liver of the 20 weeks-old db/db mice, more specifically in glycolysis and gluconeogenesis, the fructose and mannose metabolism, and the citrate cycle (Figure 3).

Regarding metabolic pathways of glycolysis and gluconeogenesis, we found three enzymes altered: fructose-1,6-bisphosphatase 1, pyruvate dehydrogenase E1 component subunit alpha, somatic form, mitochondrial and triose phosphate isomerase 1, as well as pyruvate carboxylase mitochondrial, which together with the three previous enzymes are involved in the metabolic processes of glucose (GO:0006006). Previously, it has been reported that in diabetes and obesity gluconeogenesis is damaged [15,20]. Specifically, the proteins identified as altered in the present study have also been found to be associated with damage related to diabetes in previous studies. Indeed, fructose-1,6-bisphosphatase 1 has been associated with the regulation of appetite and adiposity [21]. Moreover, the finding of decreased expression of the pyruvate carboxylase enzyme may be because the model of diabetes we used begins to lower insulin levels after 16 weeks of age [22]. Thus, the mice used in this work would be in the process of losing weight. Contrary to our results, the enzyme triose phosphate isomerase 1 was found increased in a human cell line exposed to high concentrations of glucose, but interestingly, the expression of this enzyme decreased when the glucose concentration was increased even more [10], which would be consistent with our results. Moreover, the enzyme sorbitol dehydrogenase, which is responsible for forming sorbitol from glucose or fructose, was found to have increased. This result supports the reports claiming that pericytes cultured under high glucose levels overexpress this enzyme, producing reactive oxygen species [23], which is a determining factor in the process of insulin resistance. In addition, another research group reported that sorbitol dehydrogenase is decreased in the liver of mice with caloric restriction [24], which would support our results. Also, high levels of fructose lead to insulin resistance [25]. Another enzyme found differentially expressed was pyruvate dehydrogenase E1 component subunit alpha, somatic form, a mitochondrial enzyme responsible for transforming pyruvate into acetyl-CoA. The increase in expression matches earlier reports [13]. Succinate-CoA ligase [GDP-forming] subunit beta is mitochondrial and is involved in the citric acid cycle where it converts succinate to succinyl-CoA [13], an event that may be linked to the differential expression of other proteins found in this work, and which are part of the mitochondria (GO:0005739). This result confirms the fact that mitochondria have an important role in the pathophysiology of diabetes [26].

On the other hand, proteins that participate in the metabolism of nitrogen compounds were differentially expressed. Specifically, we found arginine and proline metabolism (mmu00330), as well as glutamine family amino acid metabolic processes (GO:0009064). These are also related to the urea cycle (Figure 3). Diabetes is traditionally considered a carbohydrate disease and to a lesser extent a lipidic one. Considering that insulin is the key anabolic regulator for peripheral protein metabolism, it stands to reason that blood amino acid availability will be altered in diabetic patients, as previously reported [27,28]. With respect to the metabolic pathway of arginine and proline metabolism, three enzymes involved were discovered: carbamoyl-phosphate synthase [ammonia], mitochondrial, creatine kinase and arginase-1. This finding reinforces an earlier report released in Akt1 +/− and Akt2 −/− mice [12]. The enzyme fumaryl-acetoacetase, in addition to participating in the glutamine family amino acid metabolic process, is also involved in apoptotic mechanisms in the liver, a phenomenon widely reported in diabetes [29,30].

Oxidation–reduction processes and diabetes have been extensively studied [31]. In this case, five altered proteins were identified: trans-1,2-dihydrobenzene-1,2-diol dehydrogenase, dimethylglycine dehydrogenase, mitochondrial; peroxiredoxin-1, pyruvate dehydrogenase E1 component subunit alpha, somatic form, mitochondrial and sorbitol dehydrogenase. Controversially, in a previous study peroxiredoxin-1 overexpression was found in the same model [32]. Such effect may be due to the age difference between the two models used in the experiments. In addition to redox processes, dimethylglycine dehydrogenase transforms dimethylglycine to sarcosine, which then becomes glycine and enters the urea cycle. Interestingly, low plasma levels of dimethylglycine were significantly associated with higher blood glucose levels [33]. Therefore, our finding about down-expression of dimethylglycine dehydrogenase supports the involvement of this enzyme in the development of T2DM.

Moreover, in this study it was found that vitamin binding is altered in the diabetic mouse. This molecular function was altered in the diabetic mouse liver through the participation of proteins 2-hydroxyacyl-CoA lyase 1, dimethylglycine dehydrogenase, and pyruvate carboxylase mitochondrial. Therefore, it is suggested that to prevent deficiencies and maintain health, the majority of diabetic individuals should receive daily vitamins at recommended doses from consumption of natural food sources and fortified foods [34]. Either way, 2-hydroxyacyl-CoA lyase 1, along with glyoxalase domain-containing protein 4 have been superficially studied and so far, their relationship with diabetes or obesity is not clear.

Another process closely related to diabetes is inflammation, which is also associated with oxidative stress [35,36]. An important molecule linking the latter two processes is the mitochondrial 60 kDa heat shock protein, also called HSP60, which is overexpressed during the oxidative stress, present in diabetes [35]. This protein binds toll-like receptors of immune cells and induces the inflammatory process through NF-κB [35], a molecule that although in this work was found not differentially expressed, was indeed found increased in a previous study in this type of mouse [37].

It has been proposed that overexpression of regucalcin blocks the breakdown of both glycogen and this protein, which together with selenium-binding protein 2 are down-expressed in ten week old mouse liver db/db [14]: this can reinforce the results presented in this paper. Finally, phosphatidylethanolamine binding protein 1, also known as RKIP1 appears to have an important role in the pathophysiology of diabetes. Based on previous report which demonstrated that inactivation of this protein results in enhanced beta cell and pancreatic growth [38].

Given that one of the main characteristics of diabetes is the presence of insulin resistance [1], it is to be expected that the proteins identified in this work show some association with this characteristic. In this context, it has been reported that three of the molecules identified with differential expression, carbamoyl-phosphate synthase; dimethylglycine dehydrogenase; and CPS1, fibrinogen beta chain. Previously, they have been associated with insulin resistance, specifically simple nucleotide polymorphisms [33,39,40]. Whereas in a murine model knockout of pyruvate dehydrogenase E1 component subunit alpha, a protein found to be increased in our study, cells were found to be more sensitive to insulin [41]. As well as other proteins, such as 60 kDa heat shock protein, peroxiredoxin-1, pyruvate carboxylase and regucalcin; they have been found increased in different models of insulin resistance [32,42,43,44].

There are some previous studies that address from a proteomics approach the effect of diabetes on the liver [8,9,10,11,12,13,14]. However, there is little similarity with the observed differential protein expression in these studies. This may be due to several factors, including organisms used: human [9] and cell lines [10]; also, the study of a specific organelle such as mitochondria [8], finding specific post translational modifications: protein lysine malonylation [11] and acetylation [13], as well as different organism genetically modified [12] or different age [14]. This variety of results can be interpreted as the effect of the heterogeneity and complexity of diabetes. We must also be cautious when interpreting these results, because it cannot be excluded that the alteration of some proteins may be due to the effect of time, and not exclusively to diabetes. Nonetheless, it should be noted that the main limitation of the present study is the lack of confirmation of differentially expressed proteins directly related to diabetes and obesity, by some other technique, such as western blotting, immunohistochemistry, or enzyme-linked immunosorbent assays. In addition, it would be advisable to use of mice of different age groups. Consequently, if we take into account these considerations, the realization of these studies in the future will allow us to better understand the pathophysiology of diabetes.

Another research group conducted a bioinformatics analysis of differentially expressed genes in the liver of patients with diabetes [45]. However, the metabolic pathways found in the study and those found by our team do not match. Differences may be due to the fact that Kutmon et al. analyzed gene expression, while we analyzed protein expression, as well as different species: humans and mice, respectively [45].

## 4. Materials and Methods

### 4.1. Animals

Heterozygote non-diabetic db/+ mice (BKS.Cg-m +/+ Leprdb/OlaHsd:), all black and lean, were purchased from Harlan Laboratories (Mexico City, Mexico); they were crossbred to get the db/db mice. All animal procedures were performed in accordance with Mexican legislation, NOM-062-ZOO-1999, and with the Guide for the care and use of laboratory animals of the National Institutes of Health (NIH, Bethesda, MD, USA). Mice were kept in the animal facility of the University of Guanajuato in Leon, Mexico under standard housing conditions with water and food *ad libitum* and with a 12 h:12 h light–dark cycle. Experimental procedures were approved by the Medical Science Department Ethics Committee at the University of Guanajuato, Mexico.

### 4.2. Sample Preparation and 2D-Polyacrylamide Gel Electrophoresis

Twenty-week-old males were selected and grouped in db/db and wild type mice (*n* = 3). Mice were sacrificed by cervical dislocation and then blood levels of glucose were determined using a commercial kit (Accutrend^®^ GCT, Roche, San Francisco, CA, USA). The liver of mice was extracted and stored in: Tris/HCl 20 mM, 10 mM EDTA, 2 mM DTT, pH 7.8 and a protease inhibitor (20 mM PMSF), then stored at −70 °C until the sample was processed. Cellular proteins were obtained by a sample grinding kit (GE Healthcare, Deutsch, Switzerland) and resuspended in 200 μL of buffer (7 M urea, 4% CHAPS, 60 mM DTT, 2 mM TBP, 2 mM thiourea, 2% IPG). Protein concentrations were determined by 2D Quant Kit (GE Healthcare). Two-dimensional gel electrophoresis was performed as previously described [5]. The protein separation pattern was visualized in 2-D gels by colloidal Coomassie Brilliant Blue staining. The gels were scanned and digital images were analyzed and compared, in a GS-800 densitometer (Bio-Rad, Hercules, CA, USA) and the ImageMaster 2D Platinum 7.0 software (GE Healthcare) as previously described [14]. Spots were quantitatively compared between diabetic and normal control groups, using the liver protein samples from three diabetic mice and three control mice. Data are presented as fold change (increase or decrease) versus matched controls. Each experiment was performed in triplicate. Statistical differences between groups were calculated using ANOVA and fold changes ≥1.5 with *p* values ≤0.05 considered significant.

### 4.3. Mass Spectrometry

The spots of interest were cut, reduced, alkylated, digested, and automatically transferred to a Matrix-assisted laser desorption/ionization (MALDI) analysis target by a Proteineer SP II and DP robot using the SPcontrol 3.1.48.0 v software (Bruker Daltonics, Bremen, Germany), with the aid of a DP Chemicals 96 gel digestion kit (Bruker Daltonics, Germany) and processed in a MALDI-TOF Autoflex (Bruker Daltonics, Germany) to obtain a mass fingerprint. We performed 100 satisfactory shots in 20 shotsteps, the peak resolution threshold was set at 1500, the signal/noise ratio of tolerance was 6, and contaminants were not excluded [5]. The spectrum was annotated by the Flex Analysis 1.2 v SD1 Patch 2 (Bruker Daltonics, Germany). The search engine MASCOT was used to compare the fingerprints against the UNIPROT [46] with the following parameters: Taxon-mouse, mass tolerance of up to 200 ppm, one miss-cleavage allowed, and as the fixed modification carbamidomethyl cysteine and oxidation of methionine as the variable modification.

### 4.4. Bioinformatics Analysis

The network of proteins was constructed by Search Tool for the Retrieval of Interacting Genes/Proteins (STRING) database, which provides a critical assessment and integration of protein-protein interactions, including direct (physical) as well as indirect (functional) associations [47]. Interactions among proteins in STRING were provided with a confidence score and the confidence score more than 0.4 was defined as the cut-off criterion. To understand the relationship among differentially expressed proteins in liver of db/db mice and their interaction with other proteins, differentially expressed proteins by Gene Ontology (GO) assignment was performed using the Database for Annotation, Visualization and Integrated Discovery (DAVID) v. 6.7 [48]. Proteins were uploaded into the DAVID functional annotation tool and compared to the mouse proteome background. Only enriched pathways and GO in functional annotation tool terms with a minimum of 3-fold enrichment and a Fisher’s Exact test *p*-value < 0.05 were defined as statistically significant.

## 5. Conclusions

In this study, twenty-six differentially expressed proteins were identified in the liver of db/db diabetic mice, which are involved in various metabolic processes related to carbohydrate and nitrogen metabolism. Results that do not agree entirely with those reported previously. However, they do reflect the importance of studying each of the variants of the disease to better understand the pathophysiology and etiology to design new pharmaceutical, nutritional, and physical activity treatments to reduce their prevalence.

## Figures and Tables

**Table 1:** Proteins differentially expressed in the db/db mouse liver.

Spot Number	Protein Name	Gene Name	UniProt Accession	Mascot Score	Sequence Coverage (%)	pI	Fold Change	Function
297	60 kDa heat shock protein, mitochondrial	Hspd1	P63038	140	33	5.91	+7.1	Macromolecular assembly
302	2-hydroxyacyl-CoA lyase 1	Hacl1	Q9QXE0	68	17	5.89	+3.3	Lipid metabolism
281	Fibrinogen beta chain	Fgb	Q8K0E8	127	37	6.68	+2.5	Coagulation and immunity
233, 338	Keratin, type I cytoskeletal 18	Krt18	P05784	147	28	5.22	+5.4	Filament reorganization
123	Keratin, type II cytoskeletal 8	Krt8	P11679	205	47	5.70	+1.7	Cell structure
186	Succinyl-CoA ligase [GDP-forming] subunit beta, mitochondrial	Suclg2	Q9Z2I8	136	35	6.58	+4.2	Citrate cycle
2	Sorbitol dehydrogenase	Sord	Q64442	69	33	6.60	+1.7	Fructose metabolism
17	Fumarylacetoacetase	Fah	P35505	100	27	6.70	+2.4	Amino-acid degradation
60	Triosephosphate isomerase	Tpi1	P17751	128	33	6.90	+2.7	Gluconeogenesis
41	Phosphatidylethanolamine-binding protein 1	Pebp1	P70296	75	51	5.19	+2.2	Binds ATP
142	Arginase-1	Arg1	Q61176	91	46	6.60	+2.50	Urea cycle
416	Pyruvate dehydrogenase E1 component subunit alpha, somatic form, mitochondrial	Pdha1	P35486	118		9.40	Novo	Citrate cycle
439	Trans-1,2-dihydrobenzene-1,2-diol dehydrogenase	Dhdh	Q9DBB8	120	36	6.03	Novo	Oxidoreductase
635	Glyoxalase domain-containing protein 4	Glod4	Q9CPV4	166	48	5.28	Novo	Detoxification
623	Pyruvate carboxylase, mitochondrial	Pcx	Q05920	128	23	6.25	−4.1	Citrate cycle
672	Carbamoyl-phosphate synthase [ammonia], mitochondrial	Cps1	Q8C196	159	17	6.48	−4.6	Urea cycle
371	Dimethylglycine dehydrogenase, mitochondrial	Dmgdh	Q9DBT9	172	26	7.60	−5.6	Amine degradation
13	Protein disulfide-isomerase A3	Pdia3	P27773	124	35	5.88	−2.1	Apoptosis
479	Selenium-binding protein 2	Selenbp2	Q63836	119	38	5.78	−2.1	Protein transport
151	Fructose-1,6-bisphosphatase 1	Fbp1	Q9QXD6	88	24	6.15	−2.2	Gluconeogenesis
35	Peroxiredoxin-1	Prdx1	P35700	207	51	8.26	−6.1	Oxidoreductase
21	Proteasome subunit alpha type-2	Psma2	P49722	71	35	8.39	−2.5	Protease
312	Stress-70 protein, mitochondrial	Hspa9	P38647	98	20	5.80	D	Chaperone
492	Regucalcin	Rgn	Q64374	73	36	5.00	D	Biosynthesis vitamin c
490	Creatine kinase M-type	Ckm	P07310	100	34	6.60	D	ATP-binding

**Table 2:** Enrichment analysis of metabolic pathways and biological function of differentially expressed proteins.

	GO/Pathway Name	*p* Value	Genes
BP ^1^	GO:0006006/Glucose metabolic process	1.6 × 10^−3^	Fbp1, Pcx, Pdha1, Tpi1
BP	GO:0043603/Cellular amide metabolic process	2 × 10^−3^	Arg1, Cps1, Tpi1
BP	GO:0009064/Glutamine family amino acid metabolic process	2.4 × 10^−3^	Arg1, Cps1, Fah
BP	GO:0046395/Carboxylic acid catabolic process	8.2 × 10^−3^	Hacl1, Dmgdh, Fah
BP	GO:0055114/Oxidation-reduction process	2.5 × 10^−2^	Dhdh, Dmgdh, Prdx1, Pdha1, Sord
CC ^2^	GO:0005739/Mitochondrion	2.5 × 10^−4^	Cps1, Dmgdh, Glod4, Hspa9, Prdx1, Hspd1, Pcx, Pdha1, Suclg2
CC	GO:0005759/Mitochondrial matrix	2.3 × 10^−2^	Hspd1, Pcx, Pdha1
MF ^3^	GO:0001883/Purine nucleoside binding	1.3 × 10^−2^	Cps1, Ckm, Dmgdh, Hspa9, Pebp1, Hspd1, Pcx, Suclg2
MF	GO:0019842/Vitamin binding	1.8 × 10^−2^	Hacl1, Dmgdh, Pcx
KEGG ^4^	mmu00020/Citrate cycle (TCA cycle)	3.2 × 10^−3^	Pcx, Pdha1, Suclg2
KEGG	mmu00051/Fructose and mannose metabolism	4.6 × 10^−3^	Fbp1, Sord, Tpi1
KEGG	mmu00330/Arginine and proline metabolism	9.2 × 10^−34^	Arg1, Cps1, Ckm
KEGG	mmu00010/Glycolysis/Gluconeogenesis	1.5 × 10^−2^	Fbp1, Pdha1, Tpi1

^1^ BP: Biological Process; ^2^ CC: Cellular Component; ^3^ MF: Molecular Function; ^4^ KEEG: Kyoto Encyclopedia of Genes and Genomes Pathway.

## Abbreviations

T2DM	Type 2 diabetes mellitus
IEF	Isoelectric focusing
STRING	Search Tool for the Retrieval of Interacting Genes/Proteins
DAVID	Database for Annotation, Visualization and Integrated Discovery
GO	Gene Ontology
BP	Biological Process
CC	Cellular Component
MF	Molecular Function
HCl	Hydrochloric acid
EDTA	Ethylenediaminetetraacetic acid
DTT	1,4-Dithiothreitol
KEEG	Kyoto Encyclopedia of Genes and Genomes Pathway
PMSF	Phenylmethylsulfonyl fluoride
CHAPS	3-[(3-Cholamidopropyl) dimethylammonio]-1-propanesulfonate hydrate
TBP	tributyl phosphine
IPG	Immobilized pH gradient
ANOVA	Analysis of variance
MALDI-TOF	matrix-assisted laser desorption/ionization Time-Of-Flight
UNIPROT	Universal Protein
